# Validation of SFRP1 Promoter Hypermethylation in Plasma as a Prognostic Marker for Survival and Gemcitabine Effectiveness in Patients with Stage IV Pancreatic Adenocarcinoma

**DOI:** 10.3390/cancers13225717

**Published:** 2021-11-15

**Authors:** Benjamin Emil Stubbe, Stine Dam Henriksen, Poul Henning Madsen, Anders Christian Larsen, Henrik Bygum Krarup, Inge Søkilde Pedersen, Martin Nygård Johansen, Ole Thorlacius-Ussing

**Affiliations:** 1 Department of Gastrointestinal Surgery, Aalborg University Hospital, DK-9000 Aalborg, Denmark; stdh@rn.dk (S.D.H.); anchl@rn.dk (A.C.L.); otu@rn.dk (O.T.-U.); 2 Clinical Cancer Research Center, Aalborg University Hospital, DK-9000 Aalborg, Denmark; phm@rn.dk (P.H.M.); h.krarup@rn.dk (H.B.K.); isp@rn.dk (I.S.P.); 3 Department of Clinical Medicine, Aalborg University, DK-9000 Aalborg, Denmark; 4 Department of Molecular Diagnostics, Aalborg University Hospital, DK-9000 Aalborg, Denmark; 5 Unit of Clinical Biostatistics, Aalborg University Hospital, DK-9000 Aalborg, Denmark; martin.johansen@rn.dk

**Keywords:** biomarker, cancer, pancreatic cancer, prognostic, survival, blood-based, epigenetics, personalized therapy, DNA methylation

## Abstract

**Simple Summary:**

Pancreatic adenocarcinoma (PDAC) is a disease with an incredibly grim prognosis. Most patients die within one year of receiving the diagnosis. There are currently very few tools to help the clinician decide between treatment options and evaluate prognosis at an individual level. The aim of the current study was to assess the effect of promoter hypermethylation of secreted frizzled-related protein 1 (phSFRP1) as an independent prognostic blood-based biomarker in gemcitabine-treated patients with advanced PDAC. The study was conducted as a combined discovery and validation study. Analysis in both cohorts confirmed that patients with phSFRP1 had overall poorer survival compared to those without hypermethylation. Thus, phSFRP1 shows promise as an independent prognostic biomarker in this patient group and can hopefully aid the clinician and patient find the correct balance between quantity and quality of life.

**Abstract:**

No reliable predictive blood-based biomarkers are available for determining survival from pancreatic adenocarcinoma (PDAC). This combined discovery and validation study examines promoter hypermethylation (ph) of secreted frizzled-related protein 1 (SFRP1) in plasma-derived cell-free DNA as an independent prognostic marker for survival and Gemcitabine effectiveness in patients with stage IV PDAC. We conducted methylation-specific polymerase chain reaction analysis of the promoter region of the SFRP1 gene, based on bisulfite treatment. Survival was analyzed with Kaplan–Meier curves, log-rank test, and Cox regression. The discovery cohort included 40 patients, 25 receiving Gem. Gem-treated patients with phSFRP1 had a shorter median overall survival (mOS) (4.4 months) than unmethylated patients (11.6 months). Adjusted Cox-regression yielded a hazard rate (HR) of 3.48 (1.39–8.70). The validation cohort included 58 Gem-treated patients. Patients with phSFRP1 had a shorter mOS (3.2 months) than unmethylated patients (6.3 months). Adjusted Cox regression yielded an HR of 3.53 (1.85–6.74). In both cohorts, phSFRP1 was associated with poorer survival in Gem-treated patients. This may indicate that tumors with phSFRP1 are more aggressive and less sensitive to Gem treatment. This knowledge may facilitate tailored treatment of patients with stage IV PDAC. Further studies are planned to examine phSFRP1 in more intensive chemotherapy regimens.

## 1. Introduction

Pancreatic adenocarcinoma (PDAC) is one of the leading causes of cancer death worldwide with higher incidence rates in western countries than the rest of the world [1]. In Denmark, the median overall survival (mOS) for patients eligible for resection was 21.9 months in the period 2011–2016 compared to 10.0 months in patients undergoing the FOLFIRINOX regime with moderate to severe side effects and 5.1 months in patients treated with the less aggressive gemcitabine as monotherapy (Gem) [2]. Although more effective and intensive treatments became available in 2011 and 2013, Gem remains an important component in the palliative treatment of PDAC [3,4,5,6,7,8]. Several factors, including diagnosis at late stages, early metastasis, poor penetration of drugs into the tumor stroma, and acquired chemoresistance are potentially responsible for the very low survival rates [9,10,11]. 

Clinically useful biomarkers are desperately needed in this patient group. Currently, the only blood-based prognostic biomarker for PDAC is the serum protein-carbohydrate antigen 19-9 (CA19-9), which can aid in monitoring treatment or relapse. However, roughly 10% of the Caucasian population lacks CA19-9 production, diminishing the usefulness of this biomarker [12]. However, there has been recent advancement in the treatment sensitivity for specific subgroups of PDAC. One example is patients with a mutation in one of the homologous recombination genes PALB2, BRCA1, or BRCA2. Loss of function of these genes leads to impaired ability to repair double-stranded DNA breaks—or homologous recombination deficiency (HRD). These patients are more sensitive to DNA-damaging agents, such as platinum-based chemotherapy. Unfortunately, only 6% of the overall PDAC population harbors these changes. 

The search for prognostic biomarkers may be informed by studying the altered epigenetics of PDAC. DNA-promoter hypermethylation is the focus of the present study. DNA methylation consists of the addition of a methyl group (CH_3_) to a cytosine preceding a guanosine—a CpG dinucleotide. Hypermethylation of the promoter region can lead to low gene expression or silencing of the gene [13]. 

Low expression of the secreted frizzled-related protein 1 (SFRP1) has been linked to poor survival and suggested as a prognostic marker in patients with human biliary tract carcinoma [14]. SFRP1 hypermethylation and silencing occur early in the carcinogenesis of most cancers, including PDAC [15,16]. In renal clear cell carcinoma, promoter hypermethylation of SFRP1 has been associated with poor survival and outperforms tumor stage, tumor grade, and tumor size as an independent survival marker [17]. Likewise, SFRP1 promoter hypermethylation has been suggested as an independent prognostic factor for mOS in breast cancer [18]. Furthermore, it may predict early recurrence in the ampullary of Vater adenocarcinoma [19]. The primary mechanism of SFRP1 is that it protects against carcinogenesis by acting as an antagonist to the oncogenic Wnt/β-catenin pathway involved in both cancer development and embryonic development [15,20,21]. Activation of this pathway may be required for the initiation of PDAC [22]. Furthermore, Wnt signaling is likely critical for both the progression of this disease and outcomes of Gem [22]. A recent study linked activation of β-catenin to Gem resistance in PDAC cell lines [23]. 

To our knowledge, promoter hypermethylation of SFRP1 has not yet been examined as an independent prognostic biomarker in PDAC. The dual aim of this study was, first, to investigate promoter hypermethylation of SFRP1 in cell-free DNA as an independent prognostic marker of survival in stage IV PDAC Gem-treated patients compared to patients receiving only best supportive care (BSC); second, to validate this investigation in an external cohort of patients and examine the effect of SFRP1 promoter hypermethylation on the efficiency of Gem in stage IV PDAC patients.

## 2. Materials and Methods

### 2.1. Study Design

The current study was conducted according to the REMARK guidelines (Reporting Recommendations for Tumor Marker Prognostic Studies). The discovery cohort included patients with stage IV PDAC treated with either BSC or palliative first-line Gem. Patients were included prospectively and consecutively upon their referral to the Department of Gastrointestinal Surgery, Aalborg University Hospital, Denmark on suspicion of PDAC. Patients were included between March 2008 and February 2011. The plasma samples used in the discovery cohort originate from a study that examined the capacity of a gene panel to predict diagnosis and prognosis in patients with PDAC. Part of the data were published in an earlier study [24].

Blood samples for the validation cohort were received by courtesy of the Danish multicenter BIOPAC (Biomarkers in Patients with Pancreatic Cancer) study (NCT03311776) [25]. The BIOPAC study was approved by the local Ethics Committee (KA-20060113). Patients were included prospectively and consecutively upon referral to the Oncological Department of either Copenhagen University Hospital, Herlev, Denmark, or Rigshospitalet, Denmark. Retrospectively we defined a cohort of patients diagnosed with stage IV histologically verified PDAC, treated with palliative first-line Gem. Subsequently, blood samples on these patients were received. Patients were included between September 2011 and February 2016. Clinical data on patients were not received until analysis of blood samples had been completed. 

In both cohorts, patients were included, and blood samples were obtained before palliative treatment with Gem. All patients signed an informed consent form prior to enrollment and were above 18 years of age. All patients were chemotherapy naïve at the time of inclusion. 

### 2.2. Analytical Methods

For the discovery cohort, blood samples were centrifuged for 20 min at 4000× *g* rpm at 4 °C; subsequently, EDTA plasma was collected within 2 h after sampling and then stored at −80 °C until further analysis.

The methylation analyses were conducted by a single expert laboratory scientist, blinded to the identity of the participants. The extraction and deamination procedure of cell-free DNA has previously been detailed comprehensively by our group [24,26].

In brief, cell-free DNA was isolated from 1 mL of EDTA plasma using the EasyMAG nucleic acid purification platform (Biomeriéux, Marcy-l’Étoile, France). Bilsulfite-mediated deamination was then performed as follows: 50 μL of 10 M (NH_4_) HSO_3_-NaHSO_3_ solution was first added to 25 μL of purified DNA in PCR strips. The PCR strips were heated to 90 °C for 10 min followed by cooling to room temperature. The solution was purified and desulphonated on the EasyMAG nucleic acid purification platform (Biomeriéux). Extraction was performed using 25 μL of magnetic beads. 

A round-one polymerase chain reaction (PCR) amplification was performed in order to expand the deaminated DNA. SFRP1 was analyzed with a panel of other genes using a mix of outer methylation-specific primers (including SFRP1) (Table S1). 

Subsequently, a round-two PCR analysis was performed using the inner methylation-specific primers and methylation-specific probes, each in individual reactions [24,26].

SFRP1 was analyzed following dichotomization. An undetectable cycle threshold of the PCR was interpreted as a non-methylated SFRP1 gene promoter region, while a detectable cycle threshold of the PCR was interpreted as an SFRP1 gene with promoter hypermethylation. This method of dichotomization has previously been validated by our group [27].

For the external population, serum samples were obtained from the BIOPAC biobank by courtesy of Herlev Hospital [25]. The samples were then analyzed as described above for the discovery cohort. 

### 2.3. Statistical Methods

The patients of both the discovery cohort and the validation cohort were stratified according to SFRP1 promoter hypermethylation status and ± treatment with Gem. 

The Pearson chi-square test was used for comparison of categorical variables and while the Kruskal–Wallis test was used to compare continuous variables. Kaplan–Meier plots, log-rank tests, and Cox proportional hazard regression models were used for survival analysis. The proportional-hazards assumption was assessed with a visual inspection of the Schoenfeld residuals. Tests with *p*-values of <0.05 were considered statistically significant. 95% confidence intervals (CIs) were used. 

Survival was calculated from the date of diagnosis until the first of either death from any cause or end of follow-up. In order to avoid possible immortal time bias between the date of diagnosis and the start of treatment, Gem was treated as a time-dependent covariate. Thus, Gem-treated patients contributed survival time to the BSC group until the first day on first-line chemotherapy [28]. The end of the follow-up was 8 October 2018. 

Inverse probability of censoring weighting (IPCW) estimation of cumulative/dynamic time-dependent area under the curve (AUC) plots were computed using the “timeROC” package in R [29,30]. The 1-year concordance index was estimated using the “survival” package in R. 

Variables included in the adjusted Cox regression model were Gem-treatment, WHO performance status (PS), age > 65, and sex in the discovery cohort and WHO Performance Status (PS), age > 65, and sex in the validation cohort. 

To examine a possible association between SFRP1 promoter hypermethylation and Gem efficiency, patients were stratified into two groups by their SFRP1 promoter hypermethylation status and examined with crude Cox regression analysis. 

Calculations were carried out in either Stata v. 16, StataCorp LLC, College Station, TX, USA, or R: A language and environment for statistical computing. R Foundation for Statistical Computing, Vienna, Austria. URL: https://www.R-project.org/ (accessed on 10 September 2021).

## 3. Results

### 3.1. Results of the Discovery Cohort

The discovery cohort included 42 patients with stage IV pancreatic adenocarcinoma. Two patients were excluded due to missing data on the first day of first-line chemotherapy. Patients were stratified into four groups according to SFRP1 promoter hypermethylation status treatment with Gem/BSC. The overall proportion of SFRP1 promoter hypermethylation was 53% (21/40). Twenty-five patients (63%) were treated with first-line palliative Gem among whom SFRP1 promoter hypermethylation was detected in 36% (9/25). Fifteen patients received BSC (38%) of whom SFRP1 promoter hypermethylation was detected in 80% (12/15). Significantly more patients in the BSC group had phSFRP1 (*p*-value < 0.01). 

#### 3.1.1. Patient Characteristics

Characteristics of the four patient groups are presented in Table 1. The mean age of the entire cohort was 65 years (range 45–85 years). Mean PS was 0.76. There was a significant difference in PS across the four groups. Gem-treated patients were in a significantly better PS than patients receiving BSC (0.6 vs. 1.13, *p* = 0.01). There was no significant difference in PS according to SFRP1 promoter hypermethylation status (*p* = 0.89). The mean time from the date of diagnosis to the first day of first-line chemotherapy was 0.9 months. Curative surgery was unsuccessful in one patient. This patient did not receive adjuvant chemotherapy. There were no statistically significant differences in either age, sex, attempted curative surgery, time to first day of first-line chemotherapy, location of primary, or location of metastasis across the four groups. 

#### 3.1.2. Survival According to SFRP1 Promoter Hypermethylation

**Figure 1:**
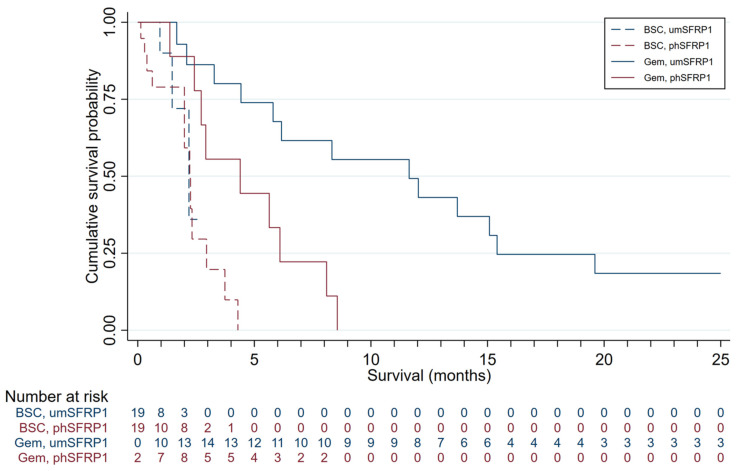
Kaplan–Meier survival distributions for patients included in the discovery cohort, grouped by SFRP1 promoter hypermethylation status and treatment. Gem-treated patients contribute survival time to the BSC group until the first day of first-line chemotherapy. Gem, patients treated with gemcitabine chemotherapy; BSC, patients receiving best supportive care; phSFRP1, patients with SFRP1 promoter hypermethylation; umSFRP1, patients without SFRP1 promoter hypermethylation. Risk table shows number of patients at risk in 1-month intervals.

Survival distributions of patients in the four groups are presented in Figure 1. The mOS was 3.3 months in the entire cohort and 6.2 months in Gem-treated patients. Gem-treated patients with SFRP1 promoter hypermethylation (*n* = 9) had an mOS of 4.4 months; those without (*n* = 16) had an mOS of 11.6 months. The survival advantage associated with not having promoter-hypermethylated SFRP1 was highly significant (log-rank test, *p* < 0.01). All patients treated with Gem succumbed to disease. Patients with phSFRP1 had an overall survival (OS) of 8.6 months, while patients with umSFRP1 had an OS of 31 months.

Patients receiving only BSC had an mOS of 2.0 months. Patients receiving BSC with an SFRP1 promoter hypermethylation (*n* = 12) had an mOS of 2.0 months; those without (*n* = 3) had an mOS of 1.5 months. No significant survival advantage was associated with lacking promoter-hypermethylated SFRP1 in these patients (log-rank test, *p* = 0.27). All patients treated with BSC succumbed to disease. Patients with phSFRP1 had an OS of 4.3 months, while patients with umSFRP1 had an OS of 2.2 months. 

Promoter hypermethylation of SFRP1 had good discriminatory capabilities for distinguishing patients who died at time t from patients who lived beyond, see Figure S1A. The accuracy was especially good at 1–2-month of follow-up, and for follow-up times 9 months and longer with AUC(t) estimates reaching 0.84. The accuracy of the model stabilized with an AUC(t) of approximately 0.8 and did not decrease substantially over time. The estimated 1-year concordance was C = 0.64.

#### 3.1.3. Crude Cox Regression Analysis

In the crude Cox regression model for OS, SFRP1 promoter hypermethylation was significantly associated with worse survival with a hazard ratio for death (HR) of 3.99 (95% CI; 1.80–8.85). Treatment with Gem and WHO PS were also significant univariate predictors of survival, while sex and age above 65 were not, see Table 2. 

#### 3.1.4. Adjusted Cox Regression Analysis

In an adjusted model, SFRP1 promoter hypermethylation was likewise significantly associated with worse survival with an HR for death of 3.48 (95% CI; 1.39–8.69). The model was adjusted for Gem-treatment, PS, age > 65, and sex. Treatment with Gem, WHO PS, and sex were also significantly associated with worse survival in the adjusted model, while age > 65 was not, see Table 2. 

#### 3.1.5. Efficiency of Gem

Patients were stratified into two groups by SFRP1 promoter hypermethylation status. The SFRP1 promoter hypermethylation group included 21 patients; 9 being treated with Gem and 12 receiving BSC. The group without SFRP1 promoter hypermethylation included 19 patients, 16 being treated with Gem and three receiving BSC.

We performed crude Cox regression on dichotomized Gem chemotherapy in the two groups.

In the SFRP1 promoter hypermethylation group, dichotomized Gem chemotherapy yielded an HR of 0.21 (95% CI; 0.07–0.67); in the group without SFRP1 promoter hypermethylation, it yielded an HR of 0.06 (95% CI; 0.01–0.38).

### 3.2. Results of the Validation Cohort

The validation cohort included 58 Gem-treated patients with stage IV pancreatic adenocarcinoma. Patients were stratified according to SFRP1 promoter hypermethylation. The overall proportion of SFRP1 hypermethylation was 50% (29/58). 

#### 3.2.1. Patient Characteristics

Characteristics of the two groups are presented in Table 3. The mean age in the entire cohort was 68 (range 46–84 years). Mean WHO PS was 0.91, significantly higher than Gem-treated patients in the discovery cohort (*p* < 0.00). There was a significant difference in the distribution of metastasis locations between the groups (*p* < 0.00). Curative surgery was attempted unsuccessfully in one patient. This patient did not receive adjuvant chemotherapy. There were no statistically significant differences in either PS, age, sex, or location of primary between the groups.

#### 3.2.2. Survival According to SFRP1 Promoter Hypermethylation

**Figure 2:**
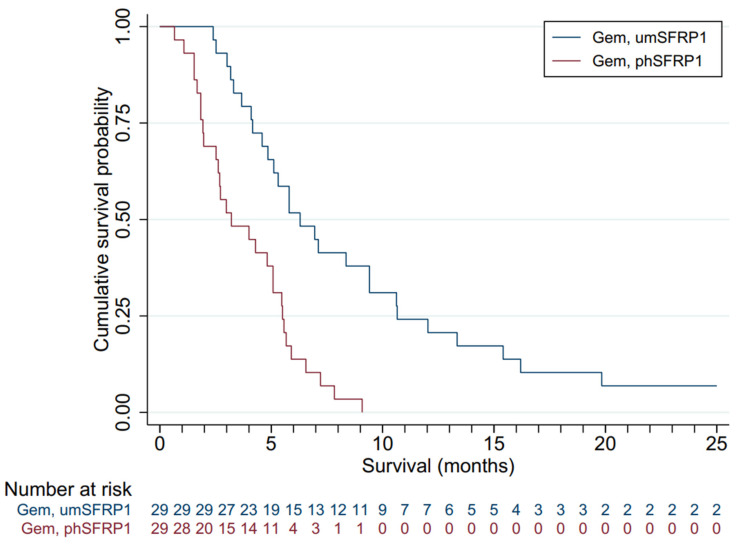
Kaplan–Meier survival distributions for patients included in the validation cohort, grouped by SFRP1 promoter hypermethylation status and treatment. Gem, gemcitabine chemotherapy; BSC, best supportive care; phSFRP1, patients with SFRP1 promoter hypermethylation; umSFRP1, patients without SFRP1 promoter hypermethylation. Risk table shows number of patients at risk in 1-month intervals.

Figure 2 presents the survival distributions of all patients in the two groups. The mOS of the entire cohort was 5.1 months. Gem-treated patients with SFRP1 promoter hypermethylation (*n* = 29) had an mOS of 3.2 months; those without (*n* = 29) had an mOS of 6.3 months. The survival advantage associated with not having promoter-hypermethylated SFRP1 was highly significant (log-rank test, *p* < 0.00). All patients succumbed to disease. Patients with phSFRP1 had an OS of 9.1 months, while patients with umSFRP1 had an OS of 63.2 months.

Promoter hypermethylation of SFRP1 had good discriminatory capabilities for distinguishing patients who died at time t from patients who lived beyond, see Figure S1B. The accuracy was especially good at 1–2-month of follow-up, and for follow-up times 9 months and longer with AUC(t) estimates reaching 0.80. The accuracy of the model stabilized with an AUC(t) of approximately 0.8 and did not decrease substantially over time. The estimated 1-year concordance was C = 0.64.

#### 3.2.3. Crude Cox Regression Analysis

Crude Cox regression of SFRP1 promoter hypermethylation was significantly associated with worse survival with an HR for death of 3.35 (95% CI; 1.82–6.16). WHO PS, sex, and age > 65 were not significant univariate predictors of survival. 

#### 3.2.4. Adjusted Cox Regression Analysis

In the adjusted model, SFRP1 promoter hypermethylation was also significantly associated with worse survival with an HR for death of 3.52 (95% CI; 1.85–6.74). The model adjusted for PS, age >65, and sex. PS, age > 65, and sex were not significant predictors of survival in the adjusted model, see Table 4.

## 4. Discussion

To the best of our knowledge, this is the first study to identify a single biomarker that may indicate both survival and the effectiveness of gemcitabine monotherapy in patients with stage IV pancreatic adenocarcinoma. The results of both our discovery cohort and validation cohort indicate that promoter hypermethylation of SFRP1 is a prognostic marker of survival in Gem-treated patients with stage IV PDAC. 

Patients with promoter-hypermethylated SFRP1 had an mOS of 4.4 months in the discovery cohort and 3.2 months in the validation cohort as compared with 11.6 months and 6.3 months, respectively, in patients without promoter-hypermethylated SFRP1. Thus, Gem-treated patients with promoter hypermethylation of SFRP1 had a much shorter mOS than what would otherwise be expected in patients with their stage of disease and type of treatment [6,7]. Crude and adjusted Cox regression analysis showed a significant association of promoter hypermethylation of SFRP1 on survival. The association was significant in both the discovery and the validation cohort. In the validation cohort, the strongest effect was observed in the adjusted analysis. These findings regarding the effect of promoter methylation of SFRP1 on survival are in accordance with previous findings in human biliary tract carcinomas and renal clear cell carcinoma [14,17].

We found a significant difference in PS between the groups in the discovery cohort. This difference is likely caused by patients receiving BSC being frailer than Gem-treated patients. We found no differences in PS according to SFRP1 promoter hypermethylation status. 

The mOS of Gem-treated patients in the validation cohort was shorter than in the discovery cohort (5.1 vs. 6.2 months). A likely explanation is a significantly poorer mean PS in the validation cohort, indicating that patients were frailer than the discovery cohort. This is likely caused by more effective and intensive palliative chemotherapy regimens becoming available in the inclusion period of the validation cohort [3,4]. As this study examined exclusively patients receiving first-line Gem, only patients unfit for the more intensive FOLFIRINOX or Gem plus nab-paclitaxel regimens were included. However, PS did not vary significantly according to SFRP1 promoter hypermethylation status in the validation cohort. This may indicate that promoter hypermethylation of SFRP1 is prognostic for survival in stage IV PDAC irrespective of PS. Promoter hypermethylation of SFRP1 was also a strong predictor of survival in the adjusted model in both cohorts. 

Time-dependent AUC(t) analysis indicates that promoter hypermethylation of SFRP1 is both an excellent short-term and long-term prognostic marker for survival. An AUC(t) of approximately 0.75 is seen in both cohorts in the first two months following diagnosis. This is followed by a drop-off in discriminatory capacity, falling to an AUC(t) of 0.61 and 0.66, respectively. This is likely caused by the frailest patients succumbing to disease. The drop-off is followed by an increase in AUC(t) to 0.84 and 0.80, respectively, in the discovery and validation study, and subsequent stabilization of an AUC(t) of approximately 0.8. This could be an indication that SFRP1 promoter hypermethylation affects survival in stage IV PDAC patients with two separate mechanisms of action. 

This study examined the association of SFRP1 promoter hypermethylation with efficiency of Gem chemotherapy in patients with stage IV pancreatic adenocarcinoma. Survival of Gem-treated patients with SFRP1 promoter hypermethylation was only marginally better than that of patients receiving BSC. This finding is striking, especially considering the significantly worse PS in the BSC group. We found no statistically significant difference in crude Cox regression analysis between the treatment groups. However, treatment with Gem was a much stronger predictor of survival in the unmethylated group than in the promoter-hypermethylated group. This may partly be explained by the small size of the groups, particularly the group without SFRP1 promoter hypermethylation who did not receive Gem. This result could not be validated as all patients in the validation cohort received Gem. Although this finding remains to be validated, it raises an important question regarding personalized medicine. If the survival of patients with SFRP1 promoter-hypermethylated stage IV PDAC is only barely improved by palliative Gem, when does quality of life become more important than quality? 

Our results indicate that promoter hypermethylation of SFRP1 is at minimum a prognostic marker for survival in Gem-treated stage IV PDAC patients. Our observations do not explain the exact mechanism of action of SFRP1 promoter hypermethylation and its relation to reduced survival. A possible mechanism of action may be an activation of the oncogenic Wnt/β-catenin pathway [15,20,22]. Previous results indicate that chemoresistance can be mediated by the Wnt/β-catenin pathway [31]. However, it is not yet clear whether the mechanism behind SFRP1 promoter hypermethylation-related chemoresistance results from a higher rate of acquired chemoresistance or a higher degree of inherent chemoresistance. Nor is it clear at exactly what point of the disease SFRP1 promoter hypermethylation exerts its effects, or whether these effects differ during the progression of the disease. How cancer cells maintain hyperactivity of Wnt/β-catenin signaling despite negative modulation also remains unclear. A recent study linked activation of β-catenin and the Erk/RAS pathway to Gem resistance in PDAC cell lines [23]. Blocking this pathway with an antagonistic molecule may be a way to sensitize PDAC cell lines to Gem [32]. SFRP1 has been suggested and examined as a possible tumor suppressor gene, as silencing may predispose to neoplastic progression [15]. Furthermore, forced re-expression of SFRP1 in several cancers induced apoptosis and suppressed cell proliferation, invasion, and transformation both in vivo and in vitro [33,34,35,36,37,38]. However, in SFRP1-null mice, no increases in the incidence of spontaneous tumor growth were observed [39]. This suggests that promoter hypermethylation of SFRP1 may be more relevant for tumor progression than for tumor genesis. This is supported by our findings of a significantly increased proportion of promoter hypermethylation of SFRP1 in the BSC group of the discovery cohort. This may indicate that PDAC tumors with promoter-hypermethylated SFRP1 are more aggressive. SFRP1 promoter hypermethylation may impact the speed with which cancer progresses or affect the tumor’s sensitivity to chemotherapy. This is in accordance with previous findings [15]. Thus, the previously examined Wnt/β-catenin pathway may be mediating chemo-resistance in SFRP1 promoter-hypermethylated PDAC. 

Another possibly relevant regulatory mechanism of SFRP1 is MicroRNAs (miRNAs). miRNAs are non-coding small RNAs that modulate gene expression at both the transcriptional and post-transcriptional level [40]. Numerous miRNAs have been linked to accelerate tumorigenesis by targeting SFRP1 [40]. The most established miRNA is miR-27a, which has been linked to inhibiting the expression of SFRP1 [40]. In gastric cancer cells, the activation of this pathway has been linked to both increased invasion, migration, and proliferation [40]. In addition, miR-27a has been linked to angiogenesis through the downregulation of BTG2 and the upregulation of VEGF in PDAC cell lines [41]. This concept is significant, as VEGF overexpression acts not only as a proangiogenic factor but also as an immune modulator by creating a permissive tumor environment as well as boosting vascular formation which leads to a poor drug response [42]. Other miRNAs have also been linked to PDAC. In a study by Zhou et al., overexpression of miR-744 was linked to enhanced cancer stem cell-like phenotypes in PDAC cell lines [43]. In addition, miRNA-940 has been linked to the regulation of SFRP1 expression in PDAC cell lines [44]. Thus, several mechanisms affect the expression of SFRP1. It is unclear whether there is any interaction between the mechanisms, but it is possible that there are several layers of SFRP1 knockout that influence prognosis in these patients. 

While some cancers have a dominant patient subgroup with viable treatment options, this is unfortunately not the case in PDAC. Though promising recent advances show increased sensitivity to platin-based treatments in patients with HDR, this subgroup amounts to only 6% of all PDAC cases [45]. Several factors play into the exceedingly poor prognosis of PDAC. One increasingly recognized aspect of the poor efficacy of treatments is the tumor microenvironment (TME). The TME is a complex interplay of immune cells, acellular stroma, soluble factors, and pancreatic stellate cells which affect drug resistance, progression of disease, and metastasis [46]. 

A possible novel approach to the treatment of PDAC might combine reactivation of SFRP1 combined with anti-angiogenesis and immunotherapy. 

Whether SFRP1 promoter hypermethylation decreases survival by itself or by affecting the efficacy of Gem requires further studies. In the context of the potential of personalized palliative treatment, it would be of great importance to identify a biomarker capable of predicting whether a patient with PDAC would benefit from Gem. 

Further studies are required to ascertain a possible predictive value of this marker. Studies are currently planned to examine a possible effect of the biomarker in patients treated with other forms of chemotherapy, as well as in the lower stages of PDAC. 

### Strengths and Limitations 

This combined discovery and validation study has a few limitations inherent to the retrospective design of the validation cohort. Missing data or selective loss of follow-up could cause selection bias. However, all patients in both cohorts were followed until death, and clinical characteristics were systematically recorded. This study is partly limited due to a relatively small sample size, which limits statistical comparison of survival between Gem-treated patients and BSC-treated patients. Another limitation of the study is the semi-quantitative method of methylation analysis. However, we have previously demonstrated there to be no significant loss of information due to the dichotomization procedure. Our team is currently developing a digital droplet PCR-based procedure that would enable quantitative analysis. To our knowledge, this is the first study to examine promoter hypermethylation of SFRP1 as an independent clinically useful blood-based prognostic biomarker for stage IV PDAC. PDAC is a complex, heterogenous malignancy that remains a colossal unmet challenge. As such, conducting research on a well-defined subgroup of patients is vital to facilitate not only personalized treatment options but also finding the right compromise between the quantity of life and a life worth living. A significant strength of the current study is the blood-based nature of the biomarker, as retrieving solid tumor biopsies can be challenging or even impossible. 

## 5. Conclusions

In conclusion, our results from both the discovery and the validation cohort indicate that promoter-hypermethylated SFRP1 is a promising independent prognostic marker for survival in Gem-treated patients with stage IV PDAC. Promoter hypermethylation of SFRP1 seems to be important in the evaluation of PDAC and its prognosis in the individual patient. Furthermore, SFRP1 expression could be interesting in relation to targeted therapy, as reversing the promoter hypermethylation of SFRP1 may be a possible way to reduce PDAC progression. Further studies are planned to evaluate a possible predictive value of the biomarker. The effects of promoter hypermethylation of SFRP1 will be examined in lower-stage PDAC and other chemotherapies. This knowledge may facilitate tailored treatment of patients with stage IV PDAC.

## Figures and Tables

**Table 1:** Characteristics on PDAC patients from the discovery cohort.

Characteristics	BSC, umSFRP	BSC, phSFRP1	Gem, umSFRP1	Gem, phSFRP1	All	*p*-Value
	(*n* = 3)	(*n* = 12)	(*n* = 16)	(*n* = 9)	(*n* = 40)	
**Age, years (mean and range)**	62 (52–71)	67 (50–85)	66 (52–74)	63 (45–78)	65 (45–85)	0.87 ^a^
**Time to palliative chemo, months (mean and range)**	-	-	0.98 (0.1–2.6)	0.73 (0–1.2)	0.89 (0–2.6)	0.65 ^a^
**Sex**						
Male	1 (33%)	6 (50%)	8 (53%)	5 (56%)	20 (51%)	0.93 ^b^
Female	2 (67%)	6 (50%)	7 (47%)	4 (44%)	19 (49%)	
**Curative surgery attempted**	0 (%)	0 (%)	1 (7%)	0 (%)	1 (3%)	0.65 ^b^
**Location of primary tumor**						
Caput	1 (33%)	4 (33%)	8 (53%)	5 (56%)	18 (46%)	0.43 ^b^
Corpus	0 (%)	3 (25%)	0 (%)	0 (%)	3 (8%)	
Cauda	1 (33%)	3 (25%)	3 (20%)	1 (11%)	8 (21%)	
Unknown	1 (33%)	2 (17%)	4 (27%)	3 (33%)	10 (26%)	
**Location of metastasis**						
Liver	2 (67%)	11 (92%)	8 (53%)	7 (78%)	28 (72%)	0.53 ^b^
Lung	0 (%)	0 (%)	0 (%)	1 (11%)	1 (3%)	
Carcinosis	1 (33%)	1 (8%)	2 (13%)	1 (11%)	5 (13%)	
Lymph nodes	0 (%)	0 (%)	1 (7%)	0 (%)	1 (3%)	
Other	0 (%)	0 (%)	1 (7%)	0 (%)	1 (3%)	
Unknown	0 (%)	0 (%)	3 (20%)	0 (%)	3 (8%)	
**WHO Performance Status**						
0	0 (%)	2 (17%)	8 (53%)	7 (78%)	17 (44%)	0.01 ^b^
1	3 (100%)	6 (50%)	2 (13%)	1 (11%)	12 (31%)	
2	0 (%)	4 (33%)	5 (33%)	1 (11%)	10 (26%)	

Gem, patients treated with gemcitabine chemotherapy; BSC, patients receiving best supportive care; phSFRP1, patients with SFRP1 promoter hypermethylation; umSFRP1, patients without SFRP1 promoter hypermethylation. ^a^ Kruskal–Wallis one-way analysis of variance. ^b^ Pearson chi-square test.

**Table 2:** Univariate and multivariate Cox regression analysis in patients from the discovery cohort.

Variable	Univariate HR (95% CI)	*p*-Value	Multivariate HR (95% CI)	*p*-Value
**SFRP1 promoter hypermethylation**				
umSFRP1	1	*p* < 0.01	1	*p* < 0.01
phSFRP1	3.99 (1.8–8.85)		3.48 (1.39–8.70)	
**Treatment**				
BSC	1	*p* < 0.01	1	0.03
Gem	0.16 (0.06–0.45)		0.29 (0.09–0.92)	
**Age above 65**				
No	1	0.24	1	0.6
Yes	0.68 (0.36–1.29)		0.83 (0.40–1.70)	
**WHO Performance Status**				
0	1	*p* < 0.01	1	*p* < 0.01
1	4.15 (1.76–9.8)		4.67 (1.80–12.13)	
2	2.01 (0.85–4.79)		1.80 (0.65–5.01)	
**Sex**				
Male	1	0.43	1	0.04
Female	1.30 (0.68–2.49)		2.16 (1.02–4.56)	

HR, hazard ratio for death; CI, confidence interval; BSC, best supportive care; Gem, gemcitabine; phSFRP1, patients with SFRP1 promoter hypermethylation; umSFRP1, patients without SFRP1 promoter hypermethylation. Treatment status analyzed as a time-varying covariate.

**Table 3:** Characteristics on PDAC patients from the validation cohort.

Characteristics	Gem, umSFRP1	Gem, phSFRP1	All	*p*-Value
	(*n* = 29)	(*n* = 29)	(*n* = 58)	
**Age, years (mean and range)**	68 (53–78)	67 (46–84)	68 (46–84)	0.46 ^a^
**Sex**				
Male	13 (45%)	16 (55%)	29 (50%)	0.50 ^b^
Female	16 (55%)	13 (45%)	29 (50%)	
**Curative surgery attempted**	1 (3%)	0 (%)	1 (2%)	0.31 ^b^
**Location of primary tumor**				
Caput	20 (69%)	18 (62%)	38 (66%)	0.53 ^b^
Corpus	4 (14%)	4 (14%)	8 (14%)	
Cauda	4 (14%)	4 (14%)	8 (14%)	
Diffuse	1 (3%)	0 (%)	1 (2%)	
Papilla	0 (%)	1 (3%)	1 (2%)	
Unknown	0 (%)	2 (7%)	2 (3%)	
**Location of metastasis**				
Liver	10 (34%)	25 (86%)	35 (60%)	*p* < 0.01 ^b^
Lung	4 (14%)	0 (%)	4 (7%)	
Liver and lung	4 (14%)	3 (10%)	7 (12%)	
Carcinosis	6 (21%)	0 (%)	6 (10%)	
Other	3 (10%)	1 (3%)	4 (7%)	
Unknown	2 (7%)	0 (%)	2 (3%)	
**WHO Performance Status**				
0	10 (34%)	4 (14%)	14 (24%)	0.18 ^b^
1	15 (52%)	20 (69%)	35 (60%)	
2	4 (14%)	5 (17%)	9 (16%)	

Gem, patients treated with gemcitabine chemotherapy; phSFRP1, patients with SFRP1 promoter hypermethylation; umSFRP1, patients without SFRP1 promoter hypermethylation. ^a^ Kruskal–Wallis one-way analysis of variance. ^b^ Pearson chi-square test.

**Table 4:** Univariate and multivariate Cox regression analysis in patients from the validation cohort.

Variable	Univariate HR (95% CI)	*p*-Value	Multivariate HR (95% CI)	*p*-Value
**SFRP1 promoter hypermethylation**				
umSFRP1	1	*p* < 0.01	1	*p* < 0.01
phSFRP1	3.35 (1.82–6.17)		3.53 (1.85–6.74)	
**Age above 65**				
No	1	0.55	1	0.96
Yes	0.84 (0.47–1.49)		0.98 (0.55–1.77)	
**WHO Performance Status**				
0	1	0.13	1	0.13
1	1.46 (0.77–2.77)		1.03 (0.52–2.05)	
2	2.44 (1.02–5.83)		2.24 (0.92–5.46)	
**Sex**				
Male	1	0.91	1	0.89
Female	1.03 (0.61–1.75)		1.04 (0.60–1.79)	

HR, hazard ratio for death; CI, confidence interval; phSFRP1, patients with SFRP1 promoter hypermethylation; umSFRP1, patients without SFRP1 promoter hypermethylation.

## Data Availability

Data are available from the corresponding author upon reasonable request and with permission from the BIOPAC team.

## Supplementary Materials

The following are available online at https://www.mdpi.com/article/10.3390/cancers13225717/s1, Figure S1: Time-dependent AUC plots; Table S1: DNA sequences for probes and primers.
