# Supplementary material for: Validation of SFRP1 Promoter Hypermethylation in Plasma as a Prognostic Marker for Survival and Gemcitabine Effectiveness in Patients with Stage IV Pancreatic Adenocarcinoma
Source: Cancers (Basel). 2021 Nov 15;13(22):5717. doi: 10.3390/cancers13225717 (PMC8616084; doi:10.3390/cancers13225717)
Supplement: Supplementary file 1 [file cancers-13-05717-s001.zip › cancers-1387869-supplementary.pdf]

Validation of SFRP1 Promoter Hypermethylation in Plasma as a Prognostic Marker for Survival and Gemcitabine Effectiveness in Patients with Stage IV Pancreatic Adenocarcinoma

Benjamin Emil Stubbe, Stine Dam Henriksen, Poul Henning Madsen, Anders Christian Larsen
Henrik Bygum Krarup, Inge Søkilde Pedersen, Martin Nygård Johansen and Ole Thorlacius-Ussing

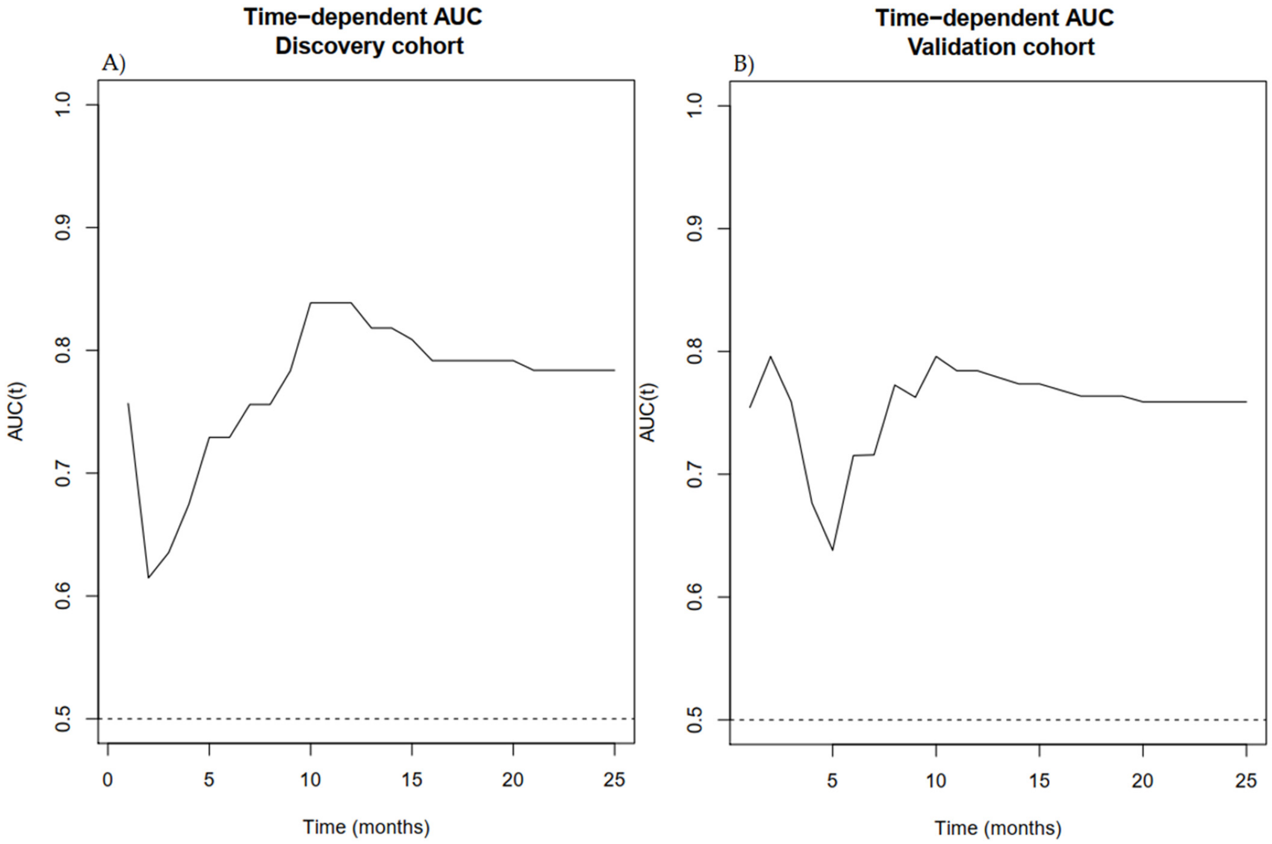

Figure S1. (**A**) IPCW estimation of cumulative/dynamic time-dependent AUC plots for patients in the discovery cohort. Accuracy of promoter hypermethylation of SFRP1 under assumption of proportional hazards. IPCW estimates of AUC(t) versus time. Estimated one-year concordance was C = 0.64 (standard error = 0.04). (**B**) IPCW estimation of cumulative/dynamic time-dependent AUC plots for patients in the validation cohort. Accuracy of promoter hypermethylation of SFRP1 under assumption of proportional hazards. IPCW estimates of AUC(t) versus time. Estimated one-year concordance was C = 0.64 (standard error = 0.04).

Table S1. DNA sequences for probes and primers.

Primer/Probe	DNA Sequence	Amplicon size
SFRP1 M1	GGA GTT GAT TGG TTG CGC	
SFRP1 M2	CGC GAC ACT AAC TCC G	90
SFRP1 M beacon	(HEX)CGC GAT G + GT T + CG + GTC G + TA ATC GCG(Dabcyl)	
SFRP1 Am	GAG GCG ATT GGT TTT CGC	
SFRP1 Bm	CGC GAC ACT AAC TCC G	121

M1; inner methylation specific forward primer for the array
M2; inner methylation specific reverse primer for the array
M beacon; methylation specific probe
Am; outer methylation specific forward primer for the nested/semi-nested PCR (Round one of PCR)
Bm; outer methylation specific reverse primer for the nested/semi-nested PCR (Round one of PCR)

+; Locked nucleic acid
